# Minutes of the Tenth Annual Meeting of the Ohio Dental College Association

**Published:** 1862-03

**Authors:** J. Taft


### Proceedings of Societies.

---

### MINUTES OF THE TENTH ANNUAL MEETING OF THE OHIO DENTAL COLLEGE ASSOCIATION.

THE Association met according to adjournment, in the lecture-room of the College, at 10 o'clock, A. M., Wednesday, Feb. 20, 1862. Dr. Bonsall, President, in the chair.

*Members present.*—Drs. Bonsall, H. R. Smith, H. A. Smith, Jas. Taylor, Joseph Richardson, and J. Taft.

The Minutes of the last meeting were read.

Dr. Jas. Taylor made some remarks in the way of explanation and suggestion in regard to the remission of the taxes.

On motion,

*Resolved,* That the Faculty be released from the payment of the taxes, and in case the tax is not remitted, the Faculty paying the same, it shall be passed to their credit at the next settlement, this arrangement to take effect from September 1st, 1861, and extend to March, 1863.

On motion of Dr. Smith,

*Resolved,* That the Secretary be requested to notify each and every stockholder of this Institution, that action will be taken at the next annual meeting to relieve the College from debt. This action may materially affect the interests of every one, and that they be urgently requested to be present, either personally or by proxy.

There seemed to be, by the conversation and suggestions of the members present, a determination that at the next annual meeting, the Institution must be relieved of debt.

The Association adjourned to meet at Dr. Taylor's office, on Thursday evening, Feb. 27th, at 7 o'clock.

Association met according to adjournment, at Dr. Taylor's office. Members present as before, with the addition of Dr. Thomas Wood.

The Treasurer presented the report for the past year.

TREASURER'S REPORT.

The Treasurer would submit the following report:

| | |
|---|---|
| Cash received—For one share of Stock, of Geo. Von Langsdorff.... | $100 00 |
| " Balance due from Dean of Faculty, up to Feb. 20, 1861,...... | 116 36 |
| " Of J. M. Lewis in Stock.... | 55 00 |
| " Of H. A. Smith.... | 65 50 |
| | $336 86 |
| CR. | |
| Paid interest on $3,000 mortgage, up to July 1, 1861.... | $150 00 |
| " on $1,000 do. up to August 1, 1861.... | 50 00 |
| " to Feb. 1, 1862.... | 50 00 |
| | $250 00 |
| Balance in Treasury.... | $86 86 |
| There is now due—Interest on $3,000 mortgage, up to Jan. 1, '62, | $150 00 |
| Floating debt, due 20th Feb. 1861.... | 247 67 |
| Interest on ditto.... | 44 58 |
| | $442 25 |
| Deduct balance in Treasury.... | 86 86 |
| | $355 39 |

*Cincinnati, Feb*, 20, 1862. JAS. TAYLOR, *Treas'r.*

Drs. H. A. Smith and H. R. Smith were appointed a committee to audit the Treasurer's account, and after due examination, reported as follows:

The auditing committee have examined the Treasurer's account, and report as follows:

| | |
|---|---|
| Amount of balance in Treasurer's hands,.... | $ 86 86 |
| " due from Faculty,.... | 400 00 |
| Insurance.... | 35 00 |
| Total,.... | $521 86 |
| Amount of Liabilities,.... | $442 25 |

H. A. SMITH,
H. R. SMITH, } *Com.*

On motion,

*Resolved*, That the Faculty be held responsible for the amount of interest on mortgages, and insurance on building, and necessary repairs, for the past year, and of present year, up to Feb. 20th, 1863. This is in consideration for use of the Ohio Dental College building for the time mentioned.

The Association then proceeded to the election of officers for the ensuing year, which resulted as follows:

*President*—CHAS. BONSALL.
1*st Vice-President*—H. R. SMITH.
2*d* " " —T. WOOD.
*Secretary*—J. TAFT.
*Treasurer*—J. TAYLOR.

Association then adjourned to meet Feb. 20th, 1863, at the Ohio Dental College. J. TAFT, *Sec'y*.

